# Transient Knockdown of RORB with Cell-Penetrating siRNA Improves Visual Function in a Proteotoxic Mouse Model of Retinitis Pigmentosa

**DOI:** 10.3390/biomedicines13102392

**Published:** 2025-09-29

**Authors:** Chanok Son, Hyo Kyung Lee, Hyoik Jang, Chul-Woo Park, Yu-sang Lee, Daehan Lim, Dong Ki Lee, Semin Lee, Hyewon Chung

**Affiliations:** 1 Department of Ophthalmology, Konkuk University College of Medicine, Seoul 05029, Republic of Korea; study0815@naver.com (C.S.); parkcw425@gmail.com (C.-W.P.); dbtkd0306@gmail.com (Y.-s.L.); 2 Department of Biomedical Engineering, College of Information and Biotechnology, Ulsan National Institute of Science and Technology (UNIST), Ulsan 44919, Republic of Korea; hklee@unist.ac.kr; 3 OliX Pharmaceuticals, Inc., Suwon 16226, Republic of Korea; hijang@olixpharma.com; 4 Department of Ophthalmology, Konkuk University Medical Center, Seoul 05029, Republic of Korea; daehan.lim0704@gmail.com

**Keywords:** retinitis pigmentosa (RP), rod photoreceptor, RORB, Rho^P23H^, proteasomal subunit

## Abstract

**Objectives:** Retinitis pigmentosa (RP) is commonly initiated by rod photoreceptor degeneration due to genetic mutations, followed by secondary cone loss and progressive blindness. Preserving rod function during the earlier stages of RP is a key therapeutic goal, as rod survival supports cone maintenance and delays vision loss. In this study, we investigated the therapeutic potential of transient knockdown of *retinoid-related orphan receptor beta* (RORB) using a cell-penetrating asymmetric small interfering RNA (cp-asiRORB) in Rho^P23H^ mice, a model of autosomal dominant RP. While the role of RORB in the adult retina remains unclear, prior studies of related nuclear receptors suggest potential involvement in proteostasis. Based on this, we hypothesized that persistent RORB expression may influence photoreceptor homeostasis under degenerative stress. **Methods:** We first optimized the cp-asiRORB design to enhance gene silencing and cellular uptake. In vitro studies were conducted under proteotoxic stress. In vivo studies involved intravitreal administration of cp-asiRORB in Rho^P23H^ mice. Furthermore, single-cell RNA sequencing of rod photoreceptors was performed. **Results:** In vitro studies demonstrated that RORB knockdown improved cell viability, reduced apoptosis, and diminished aggresome formation under proteotoxic stress. Intravitreal administration of cp-asiRORB in Rho^P23H^ mice effectively reduced RORB expression in the retina, leading to improved photoreceptor survival and preserved visual function. Single-cell RNA sequencing revealed upregulation of proteasomal subunit genes in cp-asiRORB-treated eyes, indicating enhanced proteostasis. **Conclusions:** Together, these results demonstrate that transient suppression of RORB mitigates proteotoxic stress and slows RP progression, highlighting a novel RNAi-based therapeutic strategy for retinal degeneration.

## 1. Introduction

Retinitis pigmentosa (RP) is a progressive inherited retinal degenerative disease and a leading cause of hereditary blindness, affecting approximately 1 in 3000–5000 individuals [1,2]. It is primarily characterized by the degeneration of rod photoreceptors—specialized neurons broadly distributed across the retina that are responsible for dim-light vision [3]. The loss of rods triggers apoptotic pathways, leading to secondary cone degeneration and ultimately resulting in severe visual impairment or blindness. To date, more than 3000 mutations in over 100 genes essential for rod photoreceptor development and function have been identified as causative factors of RP [1,4,5]. Despite substantial progress in elucidating the genetic basis of RP, effective medical therapies to prevent or slow rod degeneration remain limited.

The Rho^P23H^ mouse is an autosomal dominant model of RP, carrying a P23H mutation in Rhodopsin [6]. This mutation results in the accumulation of misfolded rhodopsin proteins, leading to endoplasmic reticulum (ER) stress and photoreceptor apoptosis [7,8]. This is one example of how gene mutations can give rise to misfolded proteins that disrupt cellular functions and trigger ER stress. The unfolded protein response (UPR) aims to restore homeostasis by upregulating molecular chaperones, reducing protein translation, and activating ER-associated degradation (ERAD). However, excessive accumulation of misfolded proteins can exceed the capacity of cellular degradation systems, leading to aggregate formation and aggresome sequestration, which in turn amplifies proteotoxic stress. Although proteasomal activity is initially upregulated to clear aberrant proteins, sustained stress can compromise its efficiency and impair the turnover of proteins. Ultimately, prolonged ER stress, aggregate accumulation, and proteasomal overload trigger apoptosis, contributing to photoreceptor degeneration and disease progression [9].

Nuclear hormone receptors (NRs) are a superfamily of ligand-regulated transcription factors that modulate gene expression in a tissue- and context-specific manner. Among them, retinoid-related orphan receptors (RORs)—RORA (RORα), RORB (RORβ), and RORC (RORγ)—bind DNA as monomers or dimers and play distinct physiological roles. RORA is broadly expressed in peripheral tissues, such as the heart, lungs, and adipose tissue, whereas RORC is primarily localized to immune cells [10]. In contrast, RORB shows regionally restricted expression in the central nervous system, with notably high levels in the retina, where it plays a critical role in rod photoreceptor differentiation and retinal development [11]. Previous studies have implicated RORA and RORC in the regulation of cellular stress responses and neurodegeneration, as RORA promotes ER stress and activates the UPR, whereas inhibition of RORC reduces the apoptosis of dopaminergic neurons [12,13]. Although RORB’s role in early retinal development is well established—its deletion disrupts rod photoreceptor differentiation and retinal formation—its function in the adult retina remains largely unexplored [14]. Importantly, RORB expression persists in mature photoreceptors, raising the possibility that it may serve additional roles beyond development. Given the involvement of ROR family members in stress-related signaling, we hypothesized that RORB could similarly regulate cellular stress responses in the adult retina.

Based on this rationale, we investigated whether transient knockdown of RORB could promote photoreceptor survival and ameliorate proteostasis imbalance in the Rho^P23H^ mouse model of RP. To test this hypothesis, we developed a cell-penetrating asymmetric small interfering RNA (cp-asiRNA) targeting RORB (cp-asiRORB), which enables efficient cellular uptake and potent gene silencing without the need for transfection reagents. Conjugation with cell-penetrating peptides further enhances delivery to retinal cells [15,16]. Using cp-asiRORB, we first demonstrated in vitro that RORB knockdown under proteotoxic stress improved cell viability, reduced apoptosis, and decreased aggregate formation. We then applied the same cp-asiRORB to Rho^P23H^ mice and observed enhanced photoreceptor survival and improved visual function. To elucidate the underlying mechanism, we performed single-cell RNA sequencing (scRNA-seq), which revealed the upregulation of proteasomal subunits following RORB knockdown, suggesting enhanced proteostasis. These findings highlight the therapeutic potential of targeting RORB with cp-asiRNA to alleviate proteotoxic stress and preserve visual function in RP.

## 2. Materials and Methods

### 2.1. Cell Culture

Y79 cells (ATCC, HTB-18) were cultured in RPMI-1640 medium, A549 cells (ATCC, CCL-185) in Ham’s F-12K (Kaighn’s) medium, SH-SY5Y cells (ATCC, CRL-2266) in MEM, and HEK293T (ATCC, CRL-11268) in DMEM (Welgene Inc., Gyeongsan, Republic of Korea). All media were supplemented with 10% fetal bovine serum (FBS), 100 U/mL penicillin, and 100 μg/mL streptomycin (Welgene Inc., Gyeongsan, Republic of Korea). 661W cells, a gift from Dr. Muayyad Al-Ubaidi (University of Oklahoma), were cultured in DMEM under the same conditions as described above. The cells were incubated at 37 °C in a 5% CO_2_ environment and routinely passaged every 2–3 days. Transfection was performed using 0.5 μg of plasmid (Origene Technologies, Inc., Rockville, MD, USA; RG202237) diluted in Opti-MEM I Reduced Serum Medium (Gibco, Waltham, MA, USA) with Lipofectamine 2000 (Invitrogen, Carlsbad, CA, USA). HEK293T cells were treated with 5 μM MG132 for 24 h. The medium was then replaced with fresh medium containing 500 nM cp-asiRORB, and the cells were harvested three days later for analysis.

### 2.2. siRORB Design

The sequences targeting RORB were designed to share homology with Homo sapiens, Rattus norvegicus, and Mus musculus sequences. Overall, the knockdown efficiency of the RORB-targeting constructs was validated in Y79 cells stably expressing RORB. Each strand of the designed asiRNAs was synthesized and purchased from Bioneer (Daejeon, Republic of Korea) and Dharmacon (Horizon, Lafayette, CO, USA). asiRORB was transfected into cells using Lipofectamine 2000 (Invitrogen, Carlsbad, CA, USA).

### 2.3. Synthesis of cp-asiRORB

The cp-asiRORBs were synthesized by Dharmacon (Horizon, Lafayette, CO, USA) and OliX SD (San Diego, CA, USA). A scrambled control, cp-asiSC, carrying identical chemical modifications and cholesterol conjugation as cp-asiRORB, was used as a negative control. The sense and antisense strands were mixed 1:1 in Opti-MEM or 1× PBS (Gibco, Waltham, MA, USA), heated at 95 °C for 5 min, and cooled at 37 °C for 20 min. Annealing was confirmed by 12% polyacrylamide gel electrophoresis and GelRed staining for 10 min.

### 2.4. IC50 of cp-asiRORB

SH-SY5Y and 661W cells were cultured at 1.5 × 10^5^ cells/well. The passive uptake of cp-asiRORB was assessed for 24 h at concentrations ranging from 1 to 1000 nM in SH-SY5Y cells and from 0.195 to 200 nM (2-fold increments) in 661W cells. mRNA knockdown efficiency and half maximal inhibitory concentration (IC50) values for SH-SY5Y and 661W cells were determined via qRT-PCR. The details are provided in the Supplementary Table S1.

### 2.5. RNA Extraction and qRT-PCR

Total RNA was extracted using TRI Reagent (Merck Millipore, Darmstadt, Germany) and reverse-transcribed using a high-capacity cDNA synthesis kit (Bio-Rad Laboratories, Hercules, CA, USA). cDNA was amplified using SYBR Green PCR Master Mix and analyzed using a CFX Real-Time PCR System (Bio-Rad Laboratories, Hercules, CA, USA). Primer sequences are listed in the Supplementary Table S1.

### 2.6. Cell Viability Assay

Cell viability was assessed using the CCK-8 assay (Dojindo, Kumamoto, Japan). MG132-treated cells exposed to cp-asiRORB or cp-asiSC were incubated with CCK-8 solution for 3 h, and absorbance at 450 nm was measured using a microplate reader (BioTek, Shoreline, WA, USA). Viability was calculated relative to that of the control, and the experiments were performed in triplicate.

### 2.7. Western Blot Analysis

Total proteins were extracted using RIPA buffer (Sigma-Aldrich, St. Louis, MO, USA) and quantified using the BCA assay. Equal amounts of protein were separated by SDS-PAGE and transferred to PVDF membranes (Bio-Rad Laboratories, Hercules, CA, USA). Membranes were blocked with 5% skim milk/TBS-T, incubated overnight at 4 °C with primary antibodies, and then incubated for 1 h at room temperature with HRP-conjugated secondary antibodies. The primary and secondary antibodies used are listed in the Supplementary Table S2. After incubation, the membranes were treated with an enhanced chemiluminescence (ECL) substrate and imaged using a ChemiDoc system (Bio-Rad Laboratories, Hercules, CA, USA). Western blot bands were quantified using the ImageJ 1.53 software (Fiji; National Institutes of Health, Bethesda, MD, USA).

### 2.8. Immunofluorescence

Cells were fixed with 4% paraformaldehyde (PFA) for 15 min and permeabilized with 0.1% Triton X-100 (Sigma-Aldrich, St. Louis, MO, USA) in PBS at room temperature (RT) for 10 min. After washing with PBS, the samples were blocked with 5% bovine serum albumin (BSA) in PBS for 1 h at RT. The cells were then incubated overnight at 4 °C with primary antibodies diluted in blocking buffer. A list of the antibodies used is provided in Supplementary Table S2. The next day, the samples were washed three times with PBS and incubated with Alexa Fluor-conjugated secondary antibodies (Thermo Fisher Scientific, Waltham, MA, USA) for 1 h at RT. Nuclei were stained with Hoechst 33342 (Thermo Fisher Scientific, Waltham, MA, USA) for 15 min, followed by a final wash with PBS.

For cryosectioned retinas, OCT-embedded retinal sections were fixed in 4% PFA for 15 min at room temperature, followed by permeabilization with 0.1% (*v*/*v*) Triton X-100 in PBS for 10 min at 4 °C. After washing with PBS, the sections were blocked with 3% (*v*/*v*) NGS in PBS for 1 h at RT. Following blocking, the sections were incubated at 4 °C overnight with primary antibodies that were diluted in blocking buffer. The primary antibodies used are listed in Supplementary Table S2. After incubation, unbound antibodies were removed by washing the sections three times with phosphate-buffered saline (PBS). Subsequently, the sections were treated with Alexa Fluor-conjugated secondary antibodies for 1 h at RT. Nuclei were stained with Hoechst 33342 for 15 min at RT, followed by three washes with PBS. The stained sections were then mounted with Aqua-Poly/Mount (Polysciences, Inc., Warrington, PA, USA) for imaging.

For flat-mounted retinas, trimmed eyeballs were carefully incised in the sclera using a 30-gauge sterile needle and fixed in 4% PFA on ice for 1 h. The fixed eyeballs were washed with PBS, and the anterior segments were removed from the eyeballs. The eyecups were then carefully separated to isolate the retina from the retinal pigment epithelium (RPE). The retina was permeabilized with 0.2% (*v*/*v*) Triton X-100 in PBS for 10 min, followed by blocking with 5% (*v*/*v*) NGS in PBS for 1 h at RT. Subsequent procedures, including staining and mounting, were performed as previously described. Fluorescence images were acquired using a confocal microscope (Carl Zeiss Microscopy, LLC, White Plains, NY, USA; LSM 900) with a focus on the ventral retina, and processed using ImageJ 1.53 (Fiji). Statistical analyses were performed using GraphPad Prism 8.0.2 software (GraphPad Software, San Diego, CA, USA).

### 2.9. Aggresome Detection Assay

Aggresome formation was assessed using the PROTEOSTAT Aggresome Detection Kit (Enzo Life Sciences, Inc., Farmingdale, NY, USA; ENZ-51035) according to the manufacturer’s protocol. Briefly, the cells were fixed, permeabilized on ice, and incubated with aggresome detection reagent for 30 min in the dark. Stained cells were visualized using a confocal microscope (Carl Zeiss Microscopy, LLC, White Plains, NY, USA; LSM 900), and the images were processed using ImageJ 1.53 (Fiji). Statistical analyses were performed using GraphPad Prism (version 8.0.2).

### 2.10. Experimental Animals

All animal experiments complied with the guidelines approved by the Institutional Animal Care and Use Committee (Konkuk University, KU24219) and the National Institutes of Health Guidelines for the Care and Use of Laboratory Animals. The mice were housed under a 12 h light/dark cycle at a temperature of 22 ± 2 °C and humidity of 50 ± 10%, with free access to food and water. Rho^P23H^ and rd10 mice were purchased from Jackson Laboratory (Bar Harbor, MA, USA) and bred under Specific Pathogen-Free (SPF) conditions at the Konkuk University Laboratory Animal Research Center. Genotyping was performed using PCR. Heterozygous Rho^P23H^ and homozygous rd10 mice received intravitreal injections at two weeks of age, with cp-asiRORB (1 μg/μL) administered to the right eye and cp-asiSC to the left eye. Age-matched C57BL/6J mice (DBL, Incheon, Republic of Korea) served as normal controls and received intravitreal cp-asiSC injections (1 μg/μL) in both eyes. All mice were sacrificed three weeks later for analyses. All experiments were performed using male mice to minimize possible hormonal variations, as sex-based differences in retinal responses were not the primary focus of this study.

### 2.11. Intravitreal Injection

For ocular injections, dilation was induced using 5 mg/mL phenylephrine hydrochloride and 5 mg/mL tropicamide (Hanmi Pharm, Ltd., Seoul, Republic of Korea), followed by local anesthesia with 0.5% proparacaine hydrochloride (Alcon Inc., Fort Worth, TX, USA). To induce general anesthesia for the intravitreal injections, an intraperitoneal injection was administered using a solution composed of 10 mg/mL alfaxalone (Jurox Pty Ltd., Rutherford, NSW, Australia), saline (Korean Pharmaceutical Industries Co., Ltd., Seoul, Republic of Korea), and xylazine (Rompun; Bayer AG, Leverkusen, Germany) at a 30:19:1 ratio. The anesthetized mice were placed in a lateral position, and their eyes were observed using an optical microscope (Olympus Corporation, Tokyo, Japan) at magnification. A small hole was created in the sclera using a 30-gauge sterile needle (BD Biosciences, San Jose, CA, USA). Subsequently, cp-asiRORB or cp-asiSC (1 μg/μL) was carefully injected into the vitreous chamber over 3 s using a blunt 35-gauge Hamilton micro-syringe (Hamilton Company, Reno, NV, USA). Following the injection, antibiotic ophthalmic ointment (Tarivid; Santen Pharmaceutical Co., Ltd., Osaka, Japan) was applied to the treated eyes, and the mice were maintained in a warm environment until they recovered fully from anesthesia.

### 2.12. Histopathologic Analysis

Cryosections (5 μm thick) were obtained using a cryostat (Leica CM1860; Leica Biosystems Inc., Deer Park, IL, USA) and stained with hematoxylin and eosin (H&E). The outer nuclear layer (ONL) thickness was measured from the stained sections using ImageJ 1.53 software (Fiji). Statistical analyses and graphical visualizations were performed using GraphPad Prism 8.0.2 software (GraphPad Software).

### 2.13. Electroretinography (ERG) Analysis

The mice were dark-adapted overnight for scotopic electroretinography (ERG) measurements. Before recording, the pupils were dilated using 5 mg/mL phenylephrine hydrochloride and 5 mg/mL tropicamide (Hanmi Pharm, Ltd., Seoul, Republic of Korea). Anesthesia was induced via an intraperitoneal injection of 10 mg/mL alfaxalone (Jurox Pty Ltd., Rutherford, NSW, Australia) and xylazine (Rompun; Bayer AG, Leverkusen, Germany). During the procedure, the body temperature was maintained using a 37 °C heating pad, and 2% hypromellose (Samil Pharmaceutical Co., Ltd., Seoul, Republic of Korea) was applied to prevent corneal dehydration. ERG signals were recorded using a Celeris rodent electrophysiology system (Diagnosys LLC, Lowell, MA, USA). Scotopic full-field ERG (Ganzfeld ffERG) responses, including a-wave and b-wave, were elicited by white flashes at increasing intensities (0.001 to 10 cd·s/m^2^), with three sweeps averaged for each intensity level. Light-adapted photopic ERG responses were recorded after 10 min of light adaptation using bright white flashes of increasing intensities. Following ERG recordings, ophthalmic ointment was applied to both eyes to prevent corneal damage, and the mice were returned to their home cages after full recovery on a heated mat.

### 2.14. scRNA-seq Data Generation

For scRNA-seq analysis, Rho^P23H^ mice were euthanized after intravitreal injections as described elsewhere in this study, with cp-asiRORB and cp-asiSC administered into the right and left eyes, respectively. The retinas were carefully dissected, and tissues from three mice were pooled by treatment group (right: cp-asiRORB; left: cp-asiSC) prior to single-cell dissociation. Retinal tissues were dissociated into single cells using the Papain Dissociation System (Worthington Biochemical Corporation, Lakewood, NJ, USA) according to the manufacturer’s protocol, as previously described [17].

The retinal cells were gently resuspended after being washed twice at 4 °C for 5 min at 300 g in cold Ca^2+^ and Mg^2+^ free 0.04% BSA/PBS. The samples were then counted using a LUNA-FX7 Automated Fluorescence Cell Counter (Logos Biosystems; version 1.9.6, Logos Biosystems, Gyeonggi-do, Republic of Korea) with acridine orange (AO) and propidium iodide (PI) staining (Logos Biosystems, cat no. F23001). The 10× Chromium controller and Next Gem Single Cell 3′ Reagent v3.1 kits (10× Genomics, PN-1000123) were used to prepare scRNA-seq libraries according to the 10× Chromium Single Cell 3′ v3.1 protocol (10× Genomics, document no. CG000315). To produce single-cell Gel Bead-in-emulsion (GEM), a cell suspension with a target recovery of 10,000 cells was combined with a reverse-transcription master mix. This mixture was then loaded with Single Cell 3′ Gel Beads and Partitioning Oil into a Single Cell G Chip (10× Genomics, PN-1000120). Poly adenylated mRNA from single cells was uniquely barcoded and reverse-transcribed within the GEM. Following the generation of barcoded full-length cDNA from mRNA via GEM-RT incubation, the barcoded cDNA molecules underwent amplification through PCR. For the construction of the 3′ Gene Expression Library, the enriched cDNA was subjected to a series of processes, including enzymatic fragmentation, end-repair, A-tailing, adaptor ligation, and index PCR. According to the qPCR Quantification Protocol Guide (KAPA Library Quantification Kits, Rotkreuz, Switzerland), purified libraries were quantified using qPCR and validated using an Agilent Technologies 4200 TapeStation (Agilent Technologies, Santa Clara, CA, USA). The Illumina NovaSeq platform was used to sequence the libraries.

### 2.15. scRNA-seq Data Analysis

Using the mouse reference (mm10), the fastq raw files of the two groups (cp-asiRORB and cp-asiSC) were mapped. Cell Ranger pipline (version 8.0.0; 10× Genomics Chromium, Pleasanton, CA, USA) was performed to check the quality control of raw files, and the gene expression matrix was combined. First, cells with nonzero expression of four genes related to hemoglobin (Hbb-bs/bt and Hba-a1/a2) were removed, and cells with unique gene counts of less than 300, cell counts of more than 800, and less than 10% of reads originating from mitochondrial genes were filtered. The Seurat package (version 4.3.0, Satija Lab, New York City, NY, USA) was used to analyze all data after filtering low-quality cells. A total of 33,696 genes × 25,334 cell matrices were detected. We used FindClusters with a resolution of 1.2 and 12 distinct cell types, utilizing marker genes to classify 43 clusters with the top 28 principal components based on Uniform Manifold Approximation and Projection (UMAP). Rod photoreceptor cells were divided into eight clusters with the top six principal components using FindClusters, with a resolution parameter of 0.7. Dominance analysis was performed using the R_e/o_ ratio and the cell count in each cluster. Five clusters with R_e/o_ ratios greater than 1 in the cp-asiRORB group (Clusters 0, 2, 3, 4, and 5) were called the cp-asiRORB-D group, and three clusters with R_e/o_ ratios greater than 1 in the cp-asiSC group (Clusters 1, 6, and 7) were called the cp-asiSC-D group. The Wilcoxon test was used for statistical testing, and all results in scRNA-seq analysis were implemented in R programming (version 4.1.1).

### 2.16. Statistical Analysis

Data are presented as mean ± SD from at least three independent experiments performed in triplicate. Statistical significance was assessed using an unpaired two-tailed Student’s *t*-test (two groups) or one-way analysis of variance (ANOVA) with Tukey’s test (multiple comparisons). Analyses were performed using GraphPad Prism version 8.0.2 (GraphPad Software). Significance: n.s., *p* ≥ 0.05; * *p* < 0.05; ** *p* < 0.01; *** *p* < 0.001; **** *p* < 0.0001.

## 3. Results

### 3.1. siRNA Targeting RORB Was Optimized to Improve the Knockdown Efficiency and Cellular Uptake

To evaluate the therapeutic potential of RORB knockdown, we designed and optimized siRNA sequences targeting RORB, with improved delivery and silencing properties. The sequences of 62 siRNAs that were 100% identical to both Homo sapiens and Mus musculus were designed with asymmetric configurations targeting RORB. To assess knockdown efficacy, all sequences were transfected into Y79 cells for 48 h. They were narrowed down to 13 sequences (#14, #15, #26, #42, #43, #46, #48, #49, #52, #53, #54, #55, #58) with good protein knockdown effects (Supplementary Figure S1A). Knockdown efficacy was analyzed by treating Y79 cells with the 13 sequences for 48 h, followed by assessment of both mRNA and protein expression levels to confirm the dose dependency. The results showed that asiRORB (#26) had the most dramatic dose-dependency, with 50% inhibition of protein expression at low concentrations (1 nM) (Supplementary Figure S1B,C). Based on these results, we selected the #26 sequence for chemical modification to optimize cell-penetrating asiRORB (cp-asiRORB).

**Figure 1:**
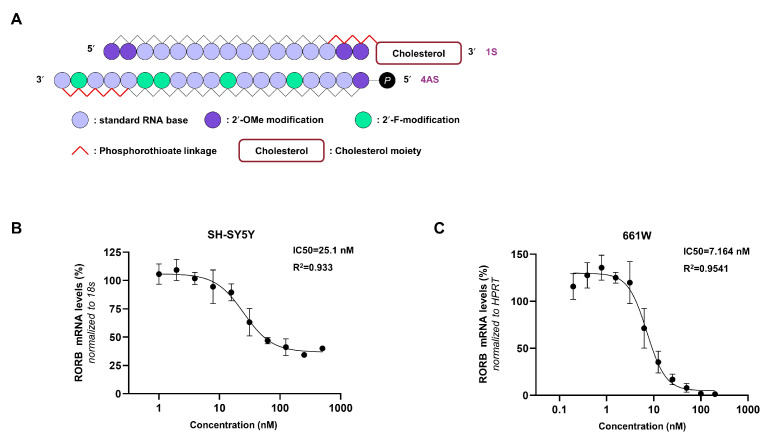
Molecular design of cell-penetrating asymmetric small interfering RNA targeting RORB (cp-asiRORB) and IC50 evaluation. (**A**) Among the designed sequences, #26 with partial modification (#26-04) showed the highest knockdown efficacy. (**B**,**C**) half maximal inhibitory concentration (IC50) curves of cp-asiRORB #26-04 across concentrations ranging from 1 to 1000 nM in SH-SY5Y (**B**) and 661W (**C**) cells. Each cell line was incubated with cp-asiRORB for 24 h, followed by qPCR analysis, normalized to 18S or hypoxanthine phosphoribosyltransferase (HPRT). Values represent mean ± SD from three independent experiments. 2′-OMe: 2′-O-Methylation, 2′-F: 2′-Fluorination.

The phosphate group at the 5′ end is essential for the guide strand of the siRNA duplex to load onto Ago2 [15,18]. To optimize this, 25 double-stranded cp-asiRORBs based on the #26 sequence were synthesized by combining five modified sense (1S-5S) and antisense (1AS-5AS) strands (Supplementary Table S3). All sequences were modified with 2′-OMe (2′-O-Methylation), 2′-F (2′-Fluorination), and phosphorothioate linkages, varying in the number and position of the 2′-modifications (Supplementary Figure S2A). Furthermore, based on the finding that cholesterol-conjugated siRNA is rapidly internalized within seconds after exposure across all cell types via EEA1-mediated endocytosis, we conjugated cholesterol to the 3′ end of the sense strand of cp-asiRORB #26-04 [19]. #26 cp-asiRORB incorporating the 1S-4AS modification (#26-04) showed the highest RORB knockdown efficiency in A549 cells (Supplementary Figure S2B–D). This modification involved replacing the phosphate backbones at the 3′ ends of both strands with phosphorothioate and adding a phosphate group to the 5′ end of the antisense strand. As a result, cp-asiRORB #26-04 had a 16mer sense strand and a 19mer antisense strand (Figure 1A). The IC50 of cp-asiRORB #26-04 was determined in human SH-SY5Y and mouse 661W cells. The IC50 values were 25.1 nM in SH-SY5Y cells and 7.164 nM in 661W cells (Figure 1B,C). These findings suggest that cp-asiRORB #26-04, with its optimized chemical modifications and cholesterol conjugation, exhibits enhanced cellular permeability and potent gene silencing efficacy. Based on its superior knockdown efficiency and cellular uptake, all subsequent experiments were conducted using cp-asiRORB #26-04 (hereafter cp-asiRORB).

### 3.2. RORB Knockdown Enhances Cell Viability and Reduces Protein Aggregation in Proteotoxic Stress Conditions

ER stress, commonly induced by the accumulation of misfolded proteins in the ER, activates UPR to maintain protein homeostasis. However, under persistent or severe stress, degradation pathways such as ERAD become impaired, resulting in proteostasis imbalance characterized by the accumulation of misfolded proteins and subsequent activation of apoptotic pathways [9]. While RORA has been shown to exacerbate ER stress and activate UPR signaling, and RORC inhibition reduces neuronal apoptosis, the role of RORB in the adult retina remains unclear.

**Figure 2:**
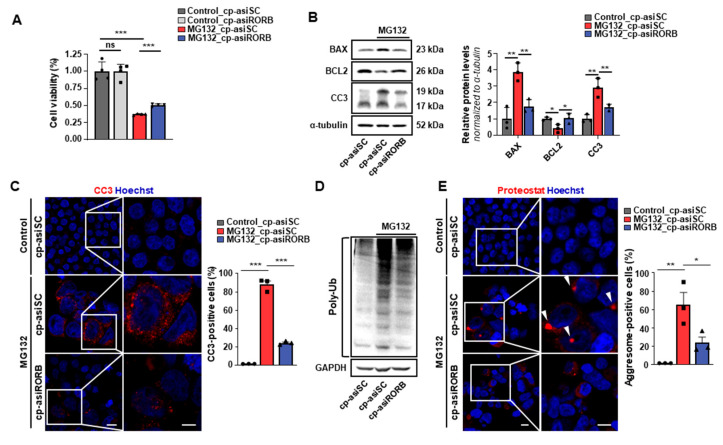
Activation of proteasomal pathways and reduction in protein aggregation following RORB knockdown. (**A**) CCK-8 assay of HEK293T cells treated with cp-asiRORB or cp-asiScramble (cp-asiSC), with or without MG132. (**B**) Quantification of Bax, Bcl2, and Cleaved Caspase-3 (CC3) by Western blot in HEK293T cells treated with MG132 followed by cp-asiRORB or cp-asiSC. *n* = 3 per group. (**C**) CC3 immunostaining in HEK293T cells treated with cp-asiRORB or cp-asiSC under MG132-induced proteotoxic stress. *n* = 3 per group. (**D**) Western blot analysis of polyubiquitinated proteins. (**E**) Quantification of protein aggresomes using PROTEOSTAT staining in HEK293T cells treated with cp-asiRORB or cp-asiSC under MG132-induced proteotoxic stress. Arrow indicates juxtanuclear aggresome formation within cells. *n* = 3 per group. The white frame indicates the region in the left panel that is magnified in the right panel. Two-sided Student’s *t*-test: *** *p* < 0.001; ** *p* < 0.01; * *p* < 0.05; ns: not significant. Scale bars: 20 μm (**C**,**E**).

To explore the potential role of RORB in proteostasis, we employed a commonly used MG132-induced proteotoxic stress model in HEK293T cells [20], transfecting cells with either cp-asiRORB or cp-asiScramble (cp-asiSC). Cells were subsequently treated with MG132, a proteasome inhibitor that induces the accumulation of misfolded protein. Cell viability was evaluated using the CCK-8 assay, which showed that cp-asiRORB treatment significantly improved cell survival under MG132-induced stress (Figure 2A).

Consistently, Western blot and immunofluorescence analyses demonstrated that cp-asiRORB attenuated MG132-induced apoptosis (Figure 2B,C).

To determine whether these protective effects were associated with enhanced protein clearance, we examined the levels of ubiquitinated proteins and aggresome formation. The accumulation of ubiquitinated proteins is a hallmark of impaired proteasomal degradation and often precedes aggregate formation. Western blot analysis revealed a reduction in polyubiquitinated proteins following cp-asiRORB treatment, suggesting enhanced proteasomal activity (Figure 2D). Furthermore, immunostaining revealed a decreased number of cells with juxtanuclear aggresomes in the cp-asiRORB-treated group (Figure 2E). These structures, formed when the proteasome system is overwhelmed, represent a compensatory mechanism for the sequestration of misfolded proteins [21]. Taken together, these results indicate that RORB knockdown enhances proteasome-dependent protein clearance and promotes cell survival under proteotoxic conditions, thereby supporting a potential role for RORB in regulating proteostasis—findings that prompted further investigation in a retinal degeneration model in vivo.

### 3.3. RORB Knockdown Restores Visual Function in Rho^P23H^ Mice

**Figure 3:**
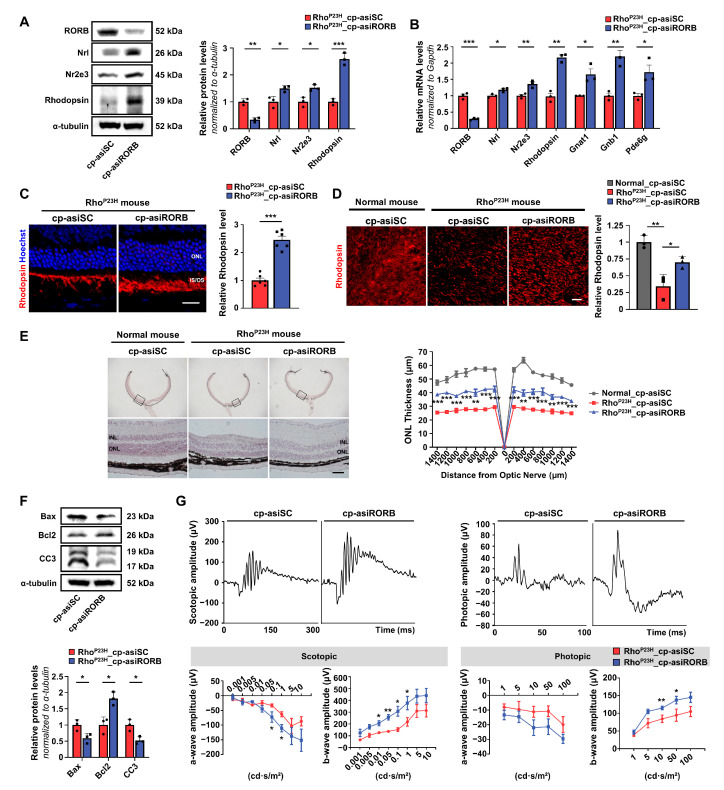
Restoration of visual function via RORB knockdown in Rho^P23H^ mice. (**A**) Western blot quantification of RORB, Nrl, Nr2e3, and Rhodopsin in retinal tissues from cp-asiRORB-treated (**right**) and cp-asiSC-treated (**left**) eyes. *n* = 3 mice per group. (**B**) qPCR of RORB and rod-specific genes in cp-asiRORB- and cp-asiSC-treated retinas. *n* = 3 mice per group. (**C**,**D**) Immunostaining of Rhodopsin in cryosectioned (**C**) and flat-mounted (**D**) retinas from cp-asiRORB-treated and cp-asiSC-treated eyes, focusing on the ventral region. *n* = 3 mice per group. (**E**) Hematoxylin and eosin (H&E) staining and outer nuclear layer (ONL) thickness measurement at 200 μm intervals from the optic nerve center. *n* = 3 mice per group. We added the following explanation to the figure legend: “The black frames indicate the regions in the top panel that are magni-fied in the bottom panel.” (**F**) Western blotting of Bax, Bcl2, and CC3 in cp-asiRORB- and cp-asiSC-treated retinal tissues. *n* = 3 mice per group. (**G**) ERG analysis of scotopic (0.001–10 cd·s/m^2^) and photopic (1–100 cd·s/m^2^) a- and b-waves in cp-asiRORB- and cp-asiSC-treated eyes. *n* = 3 mice per group. Two-sided Student’s *t*-test: *** *p* < 0.001; ** *p* < 0.01; * *p* < 0.05; ns: not significant. Scale bars: 20 μm (**C**); 50 μm (**D**,**E**). IS: Inner Segment; OS: Outer Segment.

To determine whether the in vitro effects of RORB knockdown were observed in vivo, we assessed the impact of RORB knockdown on the retinas of Rho^P23H^ transgenic mice, an established RP model. Following intravitreal injection of cp-asiRORB or cp-asiSC into 2-week-old Rho^P23H^ mice, RORB knockdown was confirmed at both the protein and mRNA levels after three weeks (Figure 3A,B). Western blot analyses showed increased expression of rod-specific genes, including Nrl, Nr2e3, and Rhodopsin, in cp-asiRORB-treated eyes compared to cp-asiSC-treated eyes (Figure 3A). mRNA analyses further confirmed increased levels of rod-differentiation genes, such as Nrl and Nr2e3, as well as phototransduction-related genes, including Rhodopsin, Gnat1, Gnb1, and Pde6g, in cp-asiRORB-treated eyes compared to the cp-asiSC-treated eyes (Figure 3B).

Consistently, immunofluorescence analysis of cryosectioned and flat-mounted retinas confirmed increased rhodopsin expression in cp-asiRORB–treated eyes compared to cp-asiSC–treated eyes (Figure 3C,D). H&E staining revealed partial restoration of the outer nuclear layer (ONL) thickness in the retinas of cp-asiRORB-treated eyes, indicating structural improvement (Figure 3E). cp-asiRORB treatment led to a significant reduction in the pro-apoptotic markers Bax and Cleaved Caspase-3, while increasing the expression of the anti-apoptotic marker Bcl2 in the retinas (Figure 3F). These results indicate that cp-asiRORB was effectively delivered in vivo, reduced apoptosis, and contributed to the survival of rod photoreceptors. Importantly, ERG analysis showed significant restoration of a- and b-wave amplitudes in cp-asiRORB–treated eyes compared with cp-asiSC–treated eyes under both scotopic and photopic conditions, indicating improved retinal function (Figure 3G).

To determine whether the protective effects of RORB knockdown observed in Rho^P23H^ mice, characterized by retinal degeneration driven by proteotoxic stress, are relevant across different RP models, we also tested cp-asiRORB in rd10 mice, a model in which retinal degeneration is primarily caused by disrupted calcium homeostasis rather than proteostasis imbalance [22,23]. Although efficient RORB knockdown was achieved in rd10 retinas, no significant morphological or functional improvements were observed, suggesting that the efficacy of cp-asiRORB may depend on the underlying pathological mechanism. Detailed data from the rd10 mice are presented in Supplementary Figure S3. These results support the idea that cp-asiRORB is particularly effective in contexts in which proteostasis disruption contributes to photoreceptor cell death, such as in the Rho^P23H^ model.

### 3.4. RORB Knockdown Upregulates Proteasomal Subunits in Rod Photoreceptors, as Revealed by Single-Cell RNA Sequencing

**Figure 4:**
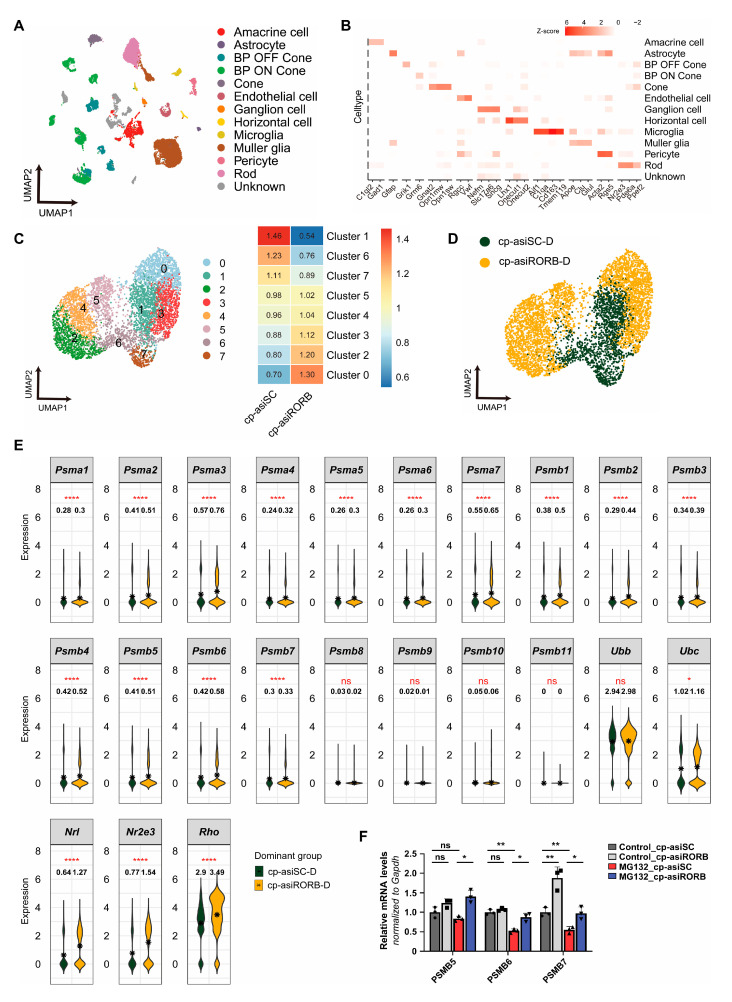
Upregulation of proteasomal subunits in rod photoreceptors following RORB knockdown, assessed by single-cell RNA sequencing. (**A**–**E**) scRNA-seq of retinal cells from Rho^P23H^ mice. (**A**) UMAP of the 12 retinal cell types. (**B**) Heatmap of marker gene expression across identified cell types. (**C**) UMAP of rod subclusters (**left**), with dominant clusters classified using the R_e/o_ ratio (**right**). (**D**) UMAP highlighting the dominant rod clusters. (**E**) Expression of rod-specific genes and proteasomal subunits in dominant clusters. (**F**) qPCR validation of proteasomal gene expression in HEK293T cells treated with cp-asiRORB or cp-asiSC under MG132 treatment. Two-sided Student’s *t*-test: n.s., *p* ≥ 0.05; * *p* < 0.05; ** *p* < 0.01; **** *p* < 0.0001. *n* = 3 per group.

Following the observed rescue of visual function in Rho^P23H^ mice, we investigated the underlying molecular mechanisms of RORB knockdown by performing scRNA–seq on retinal cells isolated from cp-asiRORB- and cp-asiSC-treated eyes. Using the 10x Genomics Chromium platform, we obtained transcriptomes from 25,334 high-quality cells. Based on canonical marker genes, 12 major retinal cell types were identified (Figure 4A,B).

To assess transcriptomic changes in rod photoreceptors, we extracted 2656 rod cells from cp-asiRORB-treated eyes and 2673 from cp-asiSC-treated eyes. These cells were grouped into eight rod-specific clusters via unsupervised classification (Figure 4C). Using the R_e/o_ ratio, which compares the relative abundance of cells between conditions within each cluster, we identified five clusters dominated by cp-asiRORB-treated cells (clusters 0, 2, 3, 4, and 5; defined as the cp-asiRORB-D group), and three clusters dominated by cp-asiSC-treated cells (clusters 1, 6, and 7; defined as the cp-asiSC-D group) (Figure 4D). Notably, the expression of rod-specific genes, including Nrl, Nr2e3, and Rho, was significantly elevated in the cp-asiRORB-D group, indicating enhanced rod photoreceptor identity and function.

To evaluate proteostasis, we compared the expression of 20 proteasomal subunit genes between dominant clusters. The majority of these genes were significantly upregulated in the cp-asiRORB-D group relative to the cp-asiSC-D group (Figure 4E), suggesting that RORB knockdown promotes proteasome-mediated protein clearance in rod photoreceptors.

To validate these transcriptomic findings, we performed qPCR in HEK293T cells treated with cp-asiRORB or cp-asiSC under MG132-induced proteotoxic stress. The mRNA levels of PSMB5 (β5), PSMB6 (β1), and PSMB7 (β2), key catalytic subunits of the 20S proteasome, were significantly elevated following cp-asiRORB treatment, further supporting proteasomal activation (Figure 4F). Together, these findings demonstrate that RORB knockdown enhances proteasome-dependent protein degradation in rod photoreceptors, thereby mitigating proteotoxic stress and contributing to the preservation of retinal structure and cellular integrity.

## 4. Discussion

This study demonstrated that siRNA-mediated knockdown of RORB preserves rod photoreceptor survival and attenuates retinal degeneration in Rho^P23H^ mice. While RORB is well established as a key transcription factor in photoreceptor development, its role in the adult retina remains largely unexplored. Our findings reveal a previously unrecognized function of RORB in regulating proteostasis in mature photoreceptors. Suppressing RORB expression mitigated proteotoxic stress and enhanced cell survival, partly by upregulating proteasome-mediated degradation pathways.

Single-cell transcriptomic analysis revealed increased expression of key proteasomal subunits (β1, β2, and β5) in rod photoreceptors following RORB knockdown. These in vivo results were consistent with our in vitro data, which showed that cp-asiRORB reduced the accumulation of polyubiquitinated proteins and aggresomes, while improving cell viability under proteotoxic stress induced by MG132. Furthermore, qPCR analysis confirmed that cp-asiRORB enhanced the expression of genes involved in proteasome function. Furthermore, our novel observation that RORB knockdown specifically elevates PSMB7 levels in non-stressed control cells suggests a more direct and specific regulatory link between RORB and this proteasomal subunit, offering an initial mechanistic clue that we plan to investigate further through additional experiments. Together, these data indicate that RORB is a negative regulator of proteasome activity and that its suppression promotes photoreceptor resilience under conditions of stress caused by protein misfolding.

To explore whether this protective mechanism extends beyond the Rho^P23H^ model, we tested cp-asiRORB in rd10 mice, which carry a Pde6b-R560C mutation that leads to photoreceptor degeneration via disrupted cGMP metabolism and calcium dysregulation, distinct from the proteotoxic stress mechanism observed in P23H-related degeneration [22,24,25,26]. In this context, cp-asiRORB did not produce a significant rescue effect. Although cp-asiRORB did not confer a protective effect in rd10 mice, this result is consistent with our central hypothesis that its efficacy may depend on the presence of proteotoxic stress as a major driver of degeneration. Importantly, proteotoxic stress is a well-documented downstream pathogenic mechanism in several forms of Inherited Retinal Diseases (IRD), not only in RHO mutations but also in others such as PRPH2, which results in proteostasis imbalance due to misfolded protein accumulation and impaired trafficking in photoreceptors [27,28]. However, therapeutic strategies directly targeting proteostasis imbalance remain limited across IRD models.

In this study, we found that transient knockdown of RORB may be clinically relevant, as it provides a safety advantage by allowing reversible modulation of the target gene, thereby minimizing long-term off-target or compensatory effects. This is particularly important in ocular diseases, where localized and repeated intravitreal or topical administrations are feasible. Our findings suggest that transient knockdown may provide sufficient therapeutic benefit during critical disease windows, while maintaining flexibility for dose adjustment or discontinuation if needed. Therefore, our findings identify RORB suppression as a targeted strategy with disease subtype selectivity, offering a framework for developing mechanism-based interventions for IRDs characterized by impaired proteostasis.

Another notable aspect of this study is the use of chemically modified, cholesterol-conjugated cell-penetrating asymmetric siRNA (cp-asiRNA), which enables efficient gene silencing without transfection reagents, viral vectors, or invasive subretinal injection [19,29,30,31]. This is important because targeting photoreceptors from the vitreous remains challenging, and most current clinical-stage therapies still rely on subretinal injection. Our results demonstrate that intravitreal delivery of cp-asiRNA effectively reduces gene expression and achieves therapeutic benefits in a genetic model of retinal degeneration, representing a clinically viable, less invasive approach to siRNA therapy.

## 5. Conclusions

In conclusion, we identified RORB as a novel regulator of proteostasis in degenerating photoreceptors and present cp-asiRORB as a promising, mechanism-based therapeutic strategy for IRDs involving proteotoxic stress. While our study focuses on the Rho^P23H^ model, these findings provide a basis for evaluating RORB-targeted interventions in broader IRD subtypes, where proteostasis plays a central role in disease progression.

## Data Availability

The raw and processed scRNA–seq data have been stored in GEO with accession number GSE291775 (link for reviewer: (https://www.ncbi.nlm.nih.gov/geo/query/acc.cgi?acc=GSE291775, accessed on 14 September 2025).

## Supplementary Materials

The following supporting information can be downloaded at: https://www.mdpi.com/article/10.3390/biomedicines13102392/s1, Figure S1: The screening of RORB-targeted asiRNA sequences. (A) Western blot images showing the screening results of 62 asiRORB sequences. All RORB-targeted asiRNA (asiRORB) sequences have 100% identity with those of mice and humans. All asiRORB sequences (1 nM) were transfected for 48 h in the Y79 cell line. (B), (C) Evaluation of dose-dependent knockdown efficacy among the top 13 asiRORB candidates, as assessed by Western blot (B) and real-time PCR (C). Among them, two asiRORB sequences (#26 and #55) showed the most significant reduction in RORB expression at both the protein and mRNA levels.3. Figure S2: Chemical modifications for screening of the most potent cell-penetrating asymmetric small interfering RNA targeting RORB (cp-asiRORB). (A) Schematic illustration of the chemical modification patterns. All sequences have differences in the numbers and positions of the 2′-modification (2′-OMe, 2′-F). (B) The selection of the most effective chemical modification combination. A total of 25 cp-asiRNAs were synthesized by combining 5 modified sense strands and 5 antisense strands, were transfected into Y79 cells for 48 h without any delivery reagent. Six cp-asiRNAs (#4, #5, #10, #24, and #25) significantly reduced RORB protein levels. (C), (D) Evaluation of dose-dependent knockdown efficacy by real-time PCR (C) and Western blot (D) using selected 5 cp-asiRORBs (#4, #5, #10, #24, #25) in A549 cell line. asiRORB-026 (10 nM) was used as a positive control. Graph values are presented as mean ± SD from two independent experiments. 2′-OMe: 2′-O-Methylation, 2`-F: 2′-Fluorination, PC: positive control (asiRORB-026, 10 nM, Transfection), NT: no treatment control, Mock: transfection reagent only, Neomycin phosphotransferase II: transfection efficiency control. Figure S3: Effects of RORB knockdown in rd10 mice. (A) RORB expression by Western blot in retinal tissues from cp-asiRORB-treated rd10 mice compared with the cp-asiScramble (cp-asiSC). (B) qPCR analysis of RORB in the retinas of cp-asiRORB-injected rd10 mice. *n* = 3 mice per group. (C) Immunostaining for Rhodopsin was performed on cryosectioned retinas from rd10 mice treated with cp-asiRORB, with imaging focused on the ventral retina. *n* = 3 mice per group. (D) Hematoxylin and eosin (H&E) staining was performed on retinal sections, and the thickness of the outer nuclear layer (ONL) was measured at 200-μm intervals from the center of the optic nerve center. *n* = 3 mice per group. (E) Expression of Bax, Bcl2, and cleaved caspase-3 (CC3) in retinal tissues of rd10 mice, as assessed by Western blot. *n* = 3 mice per group. (F) ERG analysis of visual function in cp-asiRORB- and cp-asiSC-treated rd10 mice. Scotopic and photopic a- and b-waves were recorded at the indicated light intensities. *n* = 3 per group. Two-sided Student’s *t*-test: *** *p* < 0.001, ** *p* < 0.01; ns, not significant. Scale bars: 20 μm (C), (D). Table S1: Primer sequences used for qRT-PCR experiments. Table S2: List of primary and secondary antibodies used in this study. Table S3: List of 25 Test Compounds from Combinations of 5 Sense and 5 Antisense Strands.

## Abbreviations

The following abbreviations are used in this manuscript:

RP	Retinitis pigmentosa
RORB	retinoid-related orphan receptor beta
ER	endoplasmic reticulum
UPR	unfolded protein response
ERAD	ER-associated degradation
NRs	Nuclear hormone receptors
cp-asiRNA	cell-penetrating asymmetric small interfering RNA
cp-asiRORB	cp-asiRNA targeting RORB
cp-asiSC	cp-asiRNA targeting Scramble
scRNA-seq	single-cell RNA sequencing
IRD	Inherited Retinal Diseases
PFA	paraformaldehyde
BSA	bovine serum albumin
RT	room temperature
PBS	phosphate-buffered saline
H&E	hematoxylin and eosin
ONL	outer nuclear layer
ERG	electroretinography
