# Supplementary material for: Transient Knockdown of RORB with Cell-Penetrating siRNA Improves Visual Function in a Proteotoxic Mouse Model of Retinitis Pigmentosa
Source: Biomedicines. 2025 Sep 29;13(10):2392. doi: 10.3390/biomedicines13102392 (PMC12561137; doi:10.3390/biomedicines13102392)
Supplement: Supplementary file 1 [file biomedicines-13-02392-s001.zip › revised Supplementary Figure S1.pdf]

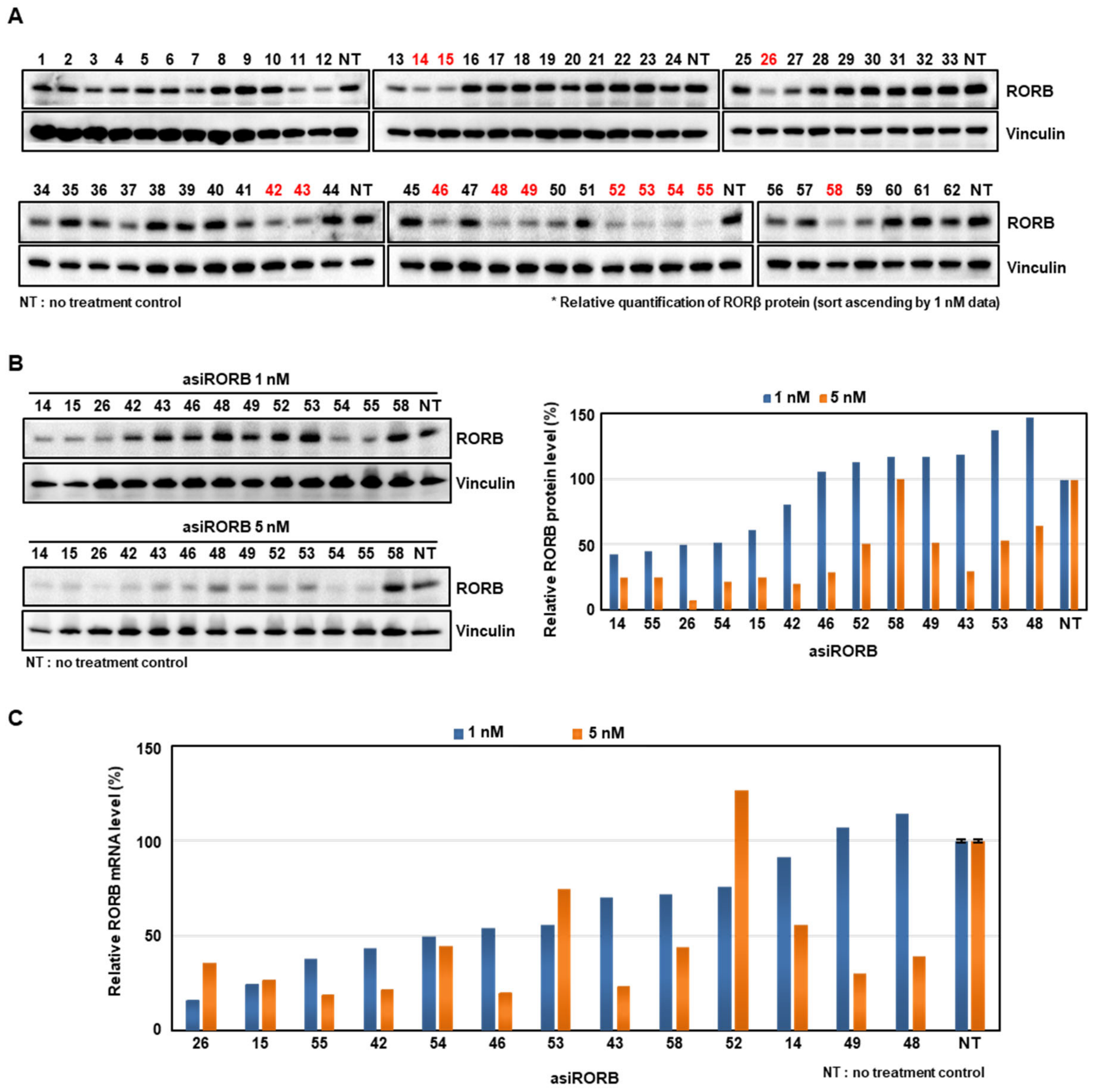

Supplementary Figure S1. The screening of RORB-targeted asiRNA sequences. (**A**) Western blot images showing the screening results of 62 asiRORB sequences. All RORB-targeted asiRNA (asiRORB) sequences have 100% identity with those of mice and humans. All asiRORB sequences (1 nM) were transfected for 48 hours in the Y79 cell line. (**B**), (**C**) Evaluation of dose-dependent knockdown efficacy among the top 13 asiRORB candidates, as assessed by western blot (**B**) and real-time PCR (**C**) Among them, two asiRORB sequences (#26 and #55) showed the most significant reduction of RORB expression at both the protein and mRNA levels.3
