# Supplementary material for: Transient Knockdown of RORB with Cell-Penetrating siRNA Improves Visual Function in a Proteotoxic Mouse Model of Retinitis Pigmentosa
Source: Biomedicines. 2025 Sep 29;13(10):2392. doi: 10.3390/biomedicines13102392 (PMC12561137; doi:10.3390/biomedicines13102392)
Supplement: Supplementary file 1 [file biomedicines-13-02392-s001.zip › Revised Supplementary Figure S2.pdf]

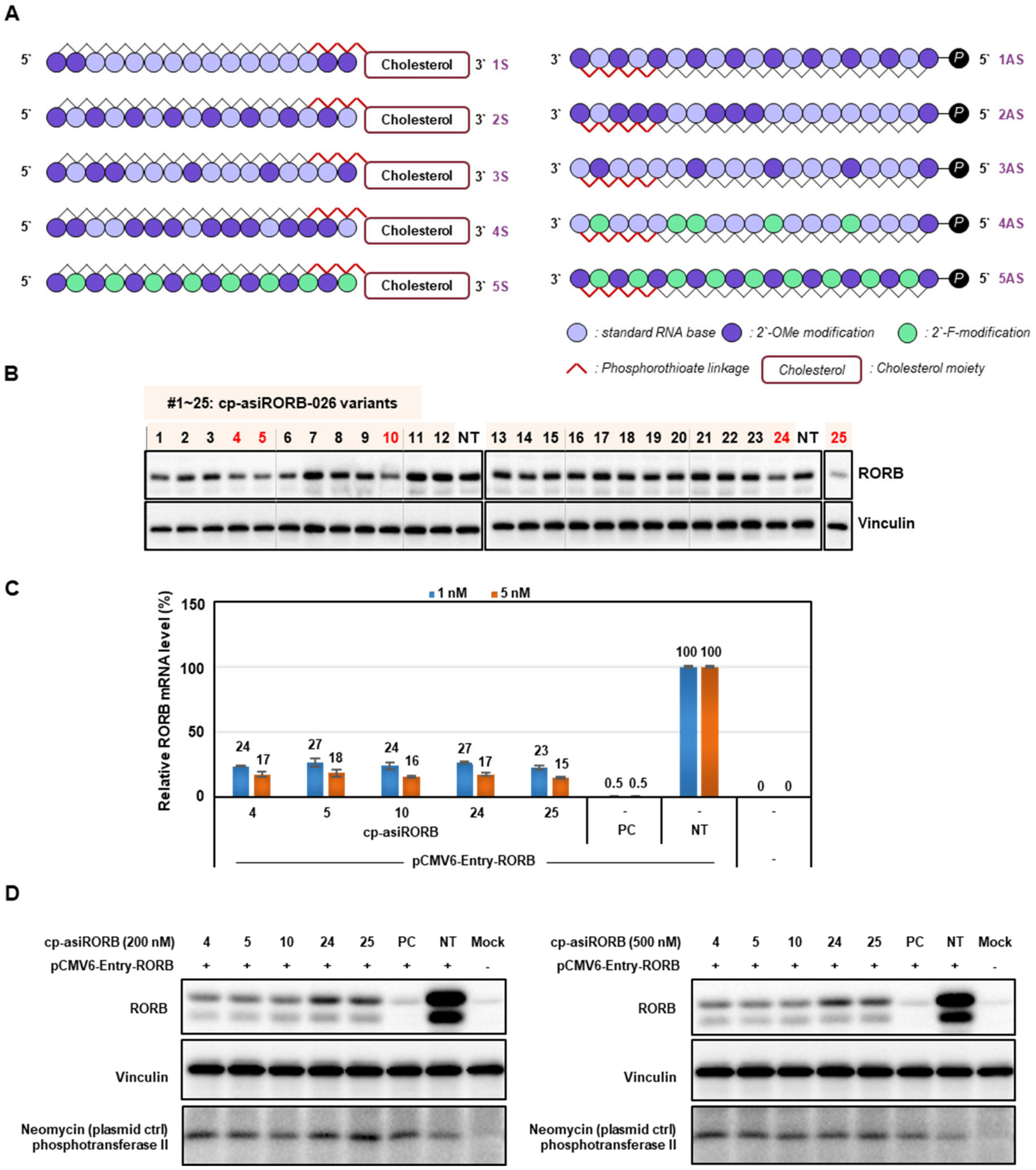

Supplementary Figure S2. Chemical modifications for screening of the most potent cell-penetrating asymmetric small interfering RNA targeting RORB (cp-asiRORB). (**A**) Schematic illustration of the chemical modification patterns. All sequences have differences in the numbers and positions of the 2'-modification (2'-OMe, 2'-F). (**B**) The selection of the most effective chemical modification combination. A total of 25 cp-asiRNAs were synthesized by combining 5 modified sense strands and 5 antisense strands, were transfected into Y79 cells for 48 hours without any delivery reagent. Six cp-asiRNAs (#4, #5, #10, #24, and #25) significantly reduced RORB protein levels. (C), (D) Evaluation of dose-dependent knockdown efficacy by real-time PCR (C) and western blot (D) using selected 5 cp-asiRORBs (#4, #5, #10, #24, #25) in A549 cell line. asiRORB-026 (10 nM) was used as a positive control. Graph values in (C) are presented as mean ± SD from two independent experiments (n=2). Data in (B) and (D) are from a single

experiment (n=1). 2'-OMe: 2'-O-Methylation, 2`-F: 2'-Fluorination, PC: positive control (asiRORB-026, 10 nM, Transfection), NT: no treatment control, Mock: transfection reagent only, Neomycin phosphotransferase II: transfection efficiency control
