# Supplementary material for: Transient Knockdown of RORB with Cell-Penetrating siRNA Improves Visual Function in a Proteotoxic Mouse Model of Retinitis Pigmentosa
Source: Biomedicines. 2025 Sep 29;13(10):2392. doi: 10.3390/biomedicines13102392 (PMC12561137; doi:10.3390/biomedicines13102392)
Supplement: Supplementary file 1 [file biomedicines-13-02392-s001.zip › revised Supplementary Figure S3.pdf]

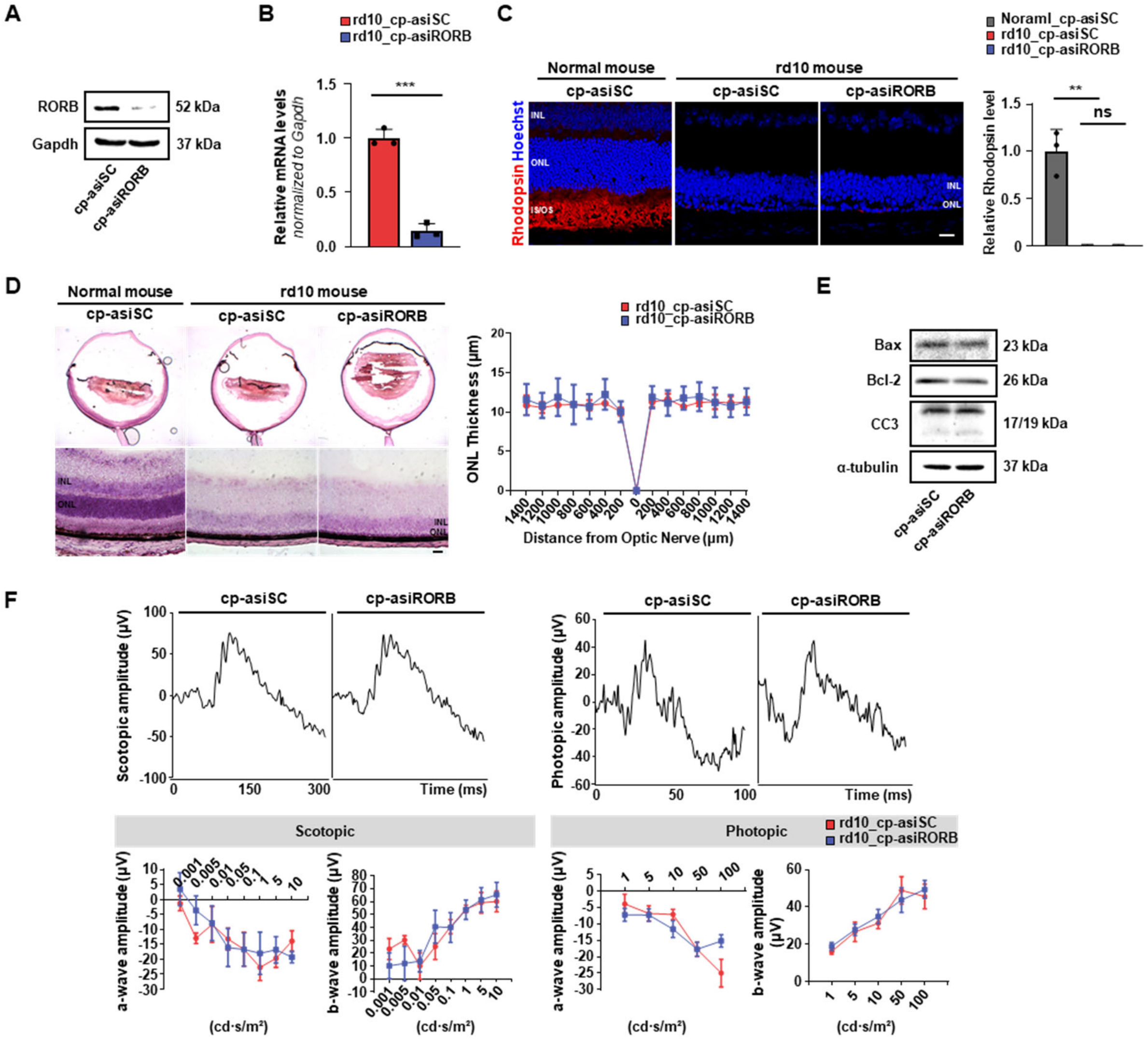

Supplementary Figure S3. Effects of RORB knockdown in rd10 mice. (**A**) RORB expression by western blot in retinal tissues from cp-asiRORB-treated rd10 mice compared with the cp-asiScramble (cp-asiSC). (**B**) qPCR analysis of RORB in the retinas of cp-asiRORB-injected rd10 mice. n = 3 mice per group. (**C**) Immunostaining for Rhodopsin was performed on cryosectioned retinas from rd10 mice treated with cp-asiRORB, with imaging focused on the ventral retina. n = 3 mice per group. (**D**) Hematoxylin and eosin (H&E) staining was performed on retinal sections, and the thickness of the outer nuclear layer (ONL) was measured at 200-μm intervals from the center of the optic nerve center. n = 3 mice per group. (**E**) Expression of Bax, Bcl2, and cleaved caspase-3 (CC3) in retinal tissues of rd10 mice, as assessed by western blot. n = 3 mice per group. (**F**) ERG analysis of visual function in cp-asiRORB- and cp-asiSC-treated rd10 mice. Scotopic and photopic a- and b-waves were recorded at indicated light intensities. n = 3 per group. Two-sided Student's t-test: *** $P < 0.001$, ** $P < 0.01$; ns, not significant. Scale bars: 20 μm (**C**, **D**).
