# Supplementary material for: Transient Knockdown of RORB with Cell-Penetrating siRNA Improves Visual Function in a Proteotoxic Mouse Model of Retinitis Pigmentosa
Source: Biomedicines. 2025 Sep 29;13(10):2392. doi: 10.3390/biomedicines13102392 (PMC12561137; doi:10.3390/biomedicines13102392)
Supplement: Supplementary file 1 [file biomedicines-13-02392-s001.zip › Revised Supplementary_Table_1.pdf]

Supplementary Material

Supplementary Table S1. Primer sequences used for qRT-PCR experiments.

Gene name	Forward (5′→3′)	Reverse (5′→3′)
RORB	TGCCCAAGTCCGAAGGTTATT	CCATGCCAGCTGATGGAGTT
HPRT	TCAGTCAACGGGGGACATAAA	GGGGCTGTACTGCTTAACCAG
Rorb	TGCCCAAGTCCGAAGGTTATT	CCATGCCAGCTGATGGAGTT
Nrl	CCAGCTGCCCGAGAGATTTT	GGTCACAGCGGGCCTTATAG
Nr2e3	TTGGAGAGGATCCAACAGGT	ATCTTGGTTCATGCCTGCTT
Rhodopsin	CATGCAATGTTCATGCGGGA	TAACCATGCGGGTGACTTCC
Gnat1	GCTTGTGGAAGGACTCGGGTAT	AACGCAACACGTCCTGCTCAGT
Gnb1	CCAGTTCTGGAGACACCACATG	TGGCTGAAGCATCACAAGCACC
Pde6g	GCGGCAAACAAGGCAGTTCAAG	CCAAGGGCAGATGACGGTGATA
Gapdh	GAGTCAACGGATTTGGTCGT	GACAAGCTTCCCGTTCTCAG
PSMB5	GTGTCCCAGAAGAGCCAGGAAT	TCTTCACCGTCTGGGAGGCAAT
PSMB6	CGTTCACTCCAGACTGGGAAAG	CGGTCGTGAATAGGTGTCAGCT
PSMB7	TTCTGGCTCCTTGGCAGCAATG	CAGGTCGTTGAAGATGCCAGCT
GAPDH	GTCTCCTCTGACTTCAACAGCG	ACCACCCTGTTGCTGTAGCCAA
**Gene name**	**TaqMan Probe**	
RORB	Hs00199445_m1, Lot#S200515-001 A04	
RN18S1	Hs03928985_g1, Lot# S190917-001 B10	
