# Supplementary material for: Transient Knockdown of RORB with Cell-Penetrating siRNA Improves Visual Function in a Proteotoxic Mouse Model of Retinitis Pigmentosa
Source: Biomedicines. 2025 Sep 29;13(10):2392. doi: 10.3390/biomedicines13102392 (PMC12561137; doi:10.3390/biomedicines13102392)
Supplement: Supplementary file 1 [file biomedicines-13-02392-s001.zip › Revised Supplementary_Table_3.pdf]

Supplementary Material

Supplementary Table S3. List of 25 Test Compounds from Combinations of 5 Sense and 5 Antisense Strands

Variant No.	Sense strand (S)	Antisense strand (AS)	Combination
Variant 1	S1	AS1	1S–1AS
Variant 2	S1	AS2	1S–2AS
Variant 3	S1	AS3	1S–3AS
Variant 4	S1	AS4	1S–4AS
Variant 5	S1	AS5	1S–5AS
Variant 6	S2	AS1	2S–1AS
Variant 7	S2	AS2	2S–2AS
Variant 8	S2	AS3	2S–3AS
Variant 9	S2	AS4	2S–4AS
Variant 10	S2	AS5	2S–5AS
Variant 11	S3	AS1	3S–1AS
Variant 12	S3	AS2	3S–2AS
Variant 13	S3	AS3	3S–3AS
Variant 14	S3	AS4	3S–4AS
Variant 15	S3	AS5	3S–5AS
Variant 16	S4	AS1	4S–1AS
Variant 17	S4	AS2	4S–2AS
Variant 18	S4	AS3	4S–3AS
Variant 19	S4	AS4	4S–4AS
Variant 20	S4	AS5	4S–5AS
Variant 21	S5	AS1	5S–1AS
Variant 22	S5	AS2	5S–2AS
Variant 23	S5	AS3	5S–3AS
Variant 24	S5	AS4	5S–4AS
Variant 25	S5	AS5	5S–5AS
