# Supplementary material for: Transient Knockdown of RORB with Cell-Penetrating siRNA Improves Visual Function in a Proteotoxic Mouse Model of Retinitis Pigmentosa
Source: Biomedicines. 2025 Sep 29;13(10):2392. doi: 10.3390/biomedicines13102392 (PMC12561137; doi:10.3390/biomedicines13102392)
Supplement: Supplementary file 1 [file biomedicines-13-02392-s001.zip › Supplementary_Table_2.pdf]

Supplementary Material

Supplementary Table S2. List of primary and secondary antibodies used in this study.

Antibodies	Dilution ratio	Cat.N	Source
RORB	1:1000	#17635-1	Proteintech
Nrl	1:500	OAAB19847	Aviva Systems Biology
Nr2e3	1:2000	14246-1-AP	Proteintech
Rhodopsin	1:1000	MAB5356	Sigma-Aldrich
Gapdh	1:2000	#2118	Cell Signaling Technology
Bax	1:500	sc-20067	Santa Cruz Biotechnology
Bcl2	1:500	sc-7382	Santa Cruz Biotechnology
Cleaved Caspase-3	1:1000	#9664	Cell Signaling Technology
FK2	1:2000	04-263	Sigma-Aldrich
α-tubulin	1:2000	#3873	Cell Signaling Technology
Goat anti-Rabbit HRP	1:10000	31460	Invitrogen
Goat anti-Mouse HRP	1:10000	31430	Invitrogen
Goat anti-Mouse IgG 555	1:400	A21424	Invitrogen
